# Predicting disease-associated substitution of a single amino acid by analyzing residue interactions

**DOI:** 10.1186/1471-2105-12-14

**Published:** 2011-01-12

**Authors:** Yizhou Li, Zhining Wen, Jiamin Xiao, Hui Yin, Lezheng Yu, Li Yang, Menglong Li

**Affiliations:** 1 Key Laboratory of Green Chemistry and Technology, Ministry of Education, College of Chemistry, Sichuan University, Chengdu 610064, PRChina

## Abstract

**Background:**

The rapid accumulation of data on non-synonymous single nucleotide polymorphisms (nsSNPs, also called SAPs) should allow us to further our understanding of the underlying disease-associated mechanisms. Here, we use complex networks to study the role of an amino acid in both local and global structures and determine the extent to which disease-associated and polymorphic SAPs differ in terms of their interactions to other residues.

**Results:**

We found that SAPs can be well characterized by network topological features. Mutations are probably disease-associated when they occur at a site with a high centrality value and/or high degree value in a protein structure network. We also discovered that study of the neighboring residues around a mutation site can help to determine whether the mutation is disease-related or not. We compiled a dataset from the Swiss-Prot variant pages and constructed a model to predict disease-associated SAPs based on the random forest algorithm. The values of total accuracy and MCC were 83.0% and 0.64, respectively, as determined by 5-fold cross-validation. With an independent dataset, our model achieved a total accuracy of 80.8% and MCC of 0.59, respectively.

**Conclusions:**

The satisfactory performance suggests that network topological features can be used as quantification measures to determine the importance of a site on a protein, and this approach can complement existing methods for prediction of disease-associated SAPs. Moreover, the use of this method in SAP studies would help to determine the underlying linkage between SAPs and diseases through extensive investigation of mutual interactions between residues.

## Background

Genetic variation is a major driving force in the evolution of organism. In individuals, specific genetic mutations such as SNPs can be deleterious and cause disease. The human genome project has yielded massive amounts of data on human SNPs, and this information can be used to further investigate human diseases. It is estimated that the human genome contains 10 million SNP sites [1]. As a major repository of human SNPs, the NCBI dbSNP database [2] contains ~25 million human entries in the release of build 130. The annotation of single nucleotide polymorphisms (SNPs) is attracting a great deal of attention. Non-synonymous SNPs (nsSNPs), also referred to as single amino acid polymorphisms (SAPs), are SNPs that cause amino acid substitutions, and these are believed to be directly related to diseases. Thus far, only a small proportion of SAPs has been associated with disease. To date, ~20,000 non-synonymous SNPs are available with explicit annotation in the Swiss-Prot database [3,4]. Therefore, it is desirable to develop effective methods for identifying disease-related amino acid substitutions[5].

Several computational models have been developed for this purpose. Evolutionary information is commonly considered to be the most important feature for such a prediction task. Based on sequence homology, an earliest predictor SIFT was developed by Ng and Henikoff [6,7]. The PANTHER database was designed based on family Hidden Markov Models (HMMs) to determine the likelihood of affecting protein function [8]. PolyPhen [9-11] showed that the selection pressure against deleterious SNPs depended on the molecular function of the proteins. Sequence/structural attributions were also incorporated in many studies. Satisfactory results were obtained by Ferrer-Costa [12] using mutation matrices, amino acid properties, and sequence potentials. By using attributions derived from other tools, an automated computational pipeline was constructed to annotate disease-associated nsSNPs [13]. Many other models have been developed based on this combination strategy [14-21]. Saunders and Baker evaluated the contributions of several structural features and evolutionary information in predicting deleterious mutations [22]. Wang and Moult undertook a detailed investigation of SNPs in which they studied the effects of the mutations on molecular function [23]. Recently, Mort *et al.*, [24] Li *et al.*, [25] and Carter *et al. *[26] functionally profiled human amino acid substitutions. They found a significant difference between deleterious and polymorphic variants in terms of both structural and functional disruption. Yue *et al. *[27-29] performed comprehensive studies on the impact of single amino acid substitutions on protein structure and stability. In these studies, stability change was also regarded as an important factor that contributed to dysfunction. Detailed studies were carried out by Reumers *et al.*, [30] and Bromberg *et al. *[31] in which the extent of the functional effect of a mutation was correlated to its effect on protein stability.

Wang *et al.*, [23] and Yue *et al. *[27] showed that the functional impacts of a mutation are closely related to its protein structural context. Recently, Alexander *et al. *[32] showed how the fold and function of a protein is altered by mutations. They observed a conformational switch between two different folds triggered by a single amino acid substitution, which directly proved the dependence of protein structure and function on amino acid interactions. Therefore, the challenge that is faced, especially when there is a lack of annotations on the functional role of a residue, is how to incorporate such useful features for detecting disease-associated mutations. To resolve this, in our study a complex network was employed to depict protein structure.

Owing to their potential for systematic analysis, complex networks have been widely used in proteomics. This method can also be used to represent a protein structure as a network (we call it protein structure network, PSN) in which the vertices are the residues and the edges are their interactions. This provides novel insight into protein folding mechanisms, stability, and function. Greene *et al.*, and Bagler *et al. *described the small-world and even scale-free [33] properties of such network, which were independent of the protein structural class [34]. Vendruscolo *et al.*, and Dokholyan *et al. *determined that a limited set of hub vertices with large connectivity plays a key role in protein folding [35-37]. In another study, hubs were defined as residues with more than four links, and these brought together different secondary structure elements that contributed to both protein folding and stability [38]. All these studies suggest that protein structure network (PSN) facilitates the systematic analysis of residue interactions both locally and globally. PSN also has the advantage of capturing the role of a residue in protein structure and function.

Using this information, Cheng *et al. *developed a solely structure-based approach named *Bongo *to predict disease-associated SAPs [39] and obtained a satisfactory positive predictive value. Their study emphasized that the functional essentiality of a site is closely correlated to its role in maintaining protein structure. Their study showed that PSN should be capable of detecting polymorphic mutations. However, their method performed poorly in detecting disease-associated mutations, which was believed to be due to the inability of *Bongo *to identify functional roles of the residue. In this study, we demonstrated that PSN can also perform well in predicting disease-associated mutations.

We carried out a comprehensive analysis on the network properties of mutations by using a dataset compiled from Swiss-Prot. We tried to determine how disease-associated variants differ from polymorphism variants in terms of network topological features. Four well-established network topological features, *degree*, *clustering coefficient*, *betweenness*, and *closeness*, were calculated based on protein structure networks and used to predict disease-associated SAPs. The neighborhood of the mutation was also investigated. These features offer a quantitative description of residue interactions. We compared their performance with that of conservation features. Finally, a model was constructed to predict disease-associated SAPs by combining network topological, conservation, and properties of neighboring residues around a mutation (environmental features) as well as several features reported in previous studies. The satisfactory performance suggested that studying residue interactions can help to distinguish disease-associated SAPs from polymorphic SAPs.

## Results

### Analysis of topological features for disease-associated and polymorphic SAPs

Four well-established network topological features, *degree*, *clustering coefficient*, *betweenness*, and *closeness*--were used to characterize disease-associated SAPs. First, an analysis was carried out to determine the extent to which disease-associated and polymorphic SAPs differ in terms of such topological features.

**Figure 1:**
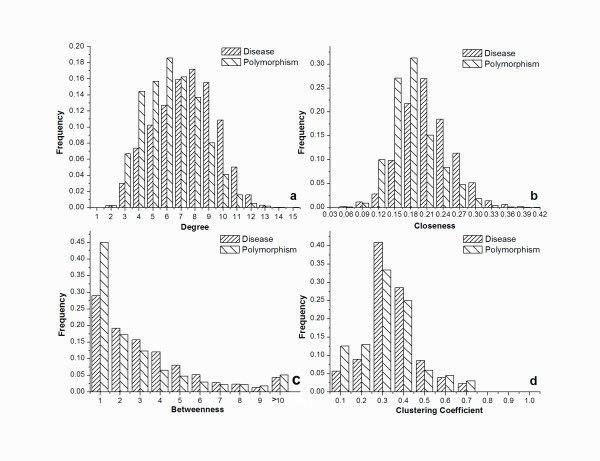
**The frequency distributions of a) degree; b) closeness; c) betweenness; and d) clustering coefficient for disease-associated and polymorphism SAPs**. In Figure 1c, the *x*-axis indicates the *betweenness *values scaled by sequence length. Here, the intervals for the frequency calculation were set to 1, 0.03, 1 and 0.1.

Figure 1 shows that the two types of SAPs differ in distributions of topological features. The frequency was specified as the ratio of SAPs with the value within [*x*,*x*+*d*) to the total number of SAPs. The *degree *reflects the number of direct interactions to an SAP. Figure 1a shows that disease-associated SAPs tend to have more neighbors than polymorphic SAPs. It is obvious that disease-associated SAPs are scored by higher *closeness *(Figure 1b). This suggests that a centrally located residue in a PSN is probably directly related to protein function. For *betweenness *and *clustering coefficient*, the distributions of disease-associated and polymorphic SAPs are less distinct. However, as shown in Figure 1c and Figure 1d, higher frequencies were detected for disease-associated SAPs in the high-scoring region.

### Comparing network topological features with conservation features

Conservation features are considered to be the most important features for predicting disease-associated SAPs. Therefore, we compared the performance of topological features, as well as environmental features with widely used conservation features in predicting disease-associated SAPs. Three feature sets were constructed: a conservation feature set (f-set 1), topological feature set (f-set 2) and neighboring environmental feature set (f-set 3). Here, f-set 1 comprises seven elements: position-specific scores and observed percentages for the wild-type and variant residues, changes in these two measures upon mutation, and the conservation score. Moreover, f-set 2 consists of the four topological features mentioned above, which are derived from the wild-type protein structure. Finally, f-set 3 consists of topological features and the conservation scores of the five most conserved neighboring residues around the SAP under study.

These three feature sets were separately used to construct prediction models based on the random forest algorithm. Details of their performances are listed in Table 1. By using the conservation feature set, ACC and MCC values of 74.1% and 0.45, respectively, were achieved. It is noteworthy that the sensitivity always appears to be better than the specificity. This is probably caused by the unbalanced ratios of disease-associated SAPs to polymorphic SAPs, as well as by the complexity of the cause of disease [23,24]. A sensitivity of ~80% suggests that conservation features can properly reflect the fragility of a residue to substitution. In the case of f-set 2, ACC and MCC were 74.6% and 0.46, respectively. Using f-set 3, an ACC and MCC values of 77.5% and 0.52, respectively, were achieved. This shows that network-based neighboring residues can properly reflect the environment around an SAP.

**Table 1:** Performance for each feature set by 5-fold cross-validation.

	Sensitivity (%)	Specificity (%)	ACC (%)	MCC
All feature set (200 ^a^,2^b^)	89.8	72.7	83.0	0.64
37-feature set (200,3)	91.0	72.3	83.6	0.65
f-set 1^c^ (100,4)	79.7	65.4	74.1	0.45
f-set 2^d^ (300,3)	81.2	63.6	74.3	0.45
f-set 3^e^ (200,1)	85.4	67.0	78.1	0.54

Optimized parameters for random forest are listed in parentheses. Detailed description of each feature set is given in the Results section.

^a ^the optimal value of *ntree *(the number of trees to be grown).

^b ^the optimal value of *mtry *(the number of variables selected to determine the decision at a node of the tree).

^c ^the conservation feature set.

^d ^the network feature set.

^e ^the environmental feature set.

### Feature evaluation

**Figure 2:**
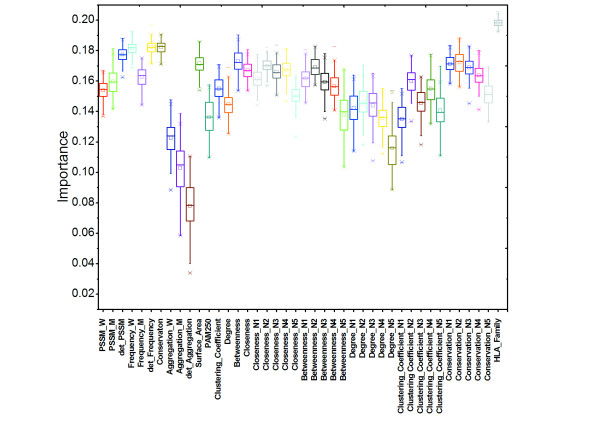
**Importance score of each feature determined by using the random forest algorithm in the R package**. Scores were averaged over 100 times. Here, suffices W, M and N indicate wild-type, mutant and environment, respectively. For environmental features, suffix numbers were used to indicate different neighboring residues. det_Frequency denotes the frequency difference between wild-type and mutant residues whereas det_PSSM denotes the PSSM score difference between wild-type and mutant residues.

Previous studies have shown that several features, such as solvent accessible area, probability of the substitution according to PAM250, aggregation propensities, and histocompatibility leukocyte antigen (HLA) family can discriminate disease-associated SAPs from polymorphic SAPs[21]. Hence, further analysis of feature importance was performed by employing the feature estimation module in the random forest package in R. As shown in Figure 2, the feature HLA family has the highest score, which is consistent with that reported by Ye *et al. *[21]. Conservation features expectedly exhibited high scores. Scores for the frequency difference and PSSM score difference between wild-type and variant residues were notably higher than those for these two features themselves. This indicates that a position would tolerate alteration between similar residues without having a marked influence on the local structure. Interestingly, the score of *closeness *is not higher than that of *betweenness*, although the results from our above analysis showed a significant difference in its distribution between disease-associated SAPs and polymorphic SAPs. As reported earlier [40], this feature is well correlated with conservations and could be the reason for its performance in this step. The environmental features were observed informative for the prediction, of which the *closeness *and *conservation *were even top ranked. This suggests that interactions between residues are crucial for the protein structure. Moreover, it was also observed that the topologically important neighbors would be more conserved. In this sense, these network-based features can reflect the structural/functional importance of residues.

### Performance of our model for prediction of disease-associated SAPs

Using all the features, we constructed a model to predict disease-associated SAPs based on a random forest algorithm. ACC and MCC values of 83.0% and 0.64, respectively, were achieved using this method with 5-fold cross-validation (Table 1). The corresponding sensitivity and specificity were 89.8% and 72.7%, respectively. When using the top-ranked 37 features in the last evaluation step (37-feature set), the performance would be slightly improved. The ACC reached 83.6% while the sensitivity improved by ~1.2%. For further evaluation, an independent dataset was used to test the method (Table 2). The sensitivity and specificity were 86.6% and 71.9%, respectively, when all features were used. The corresponding ACC and MCC values were 80.8% and 0.59, respectively. For the 37-feature set, a sensitivity of 87.3% and specificity of 72.1% were achieved. The corresponding ACC and MCC values were 81.3% and 0.60, respectively. We also tried the support vector machine algorithm on our dataset, which gave a lower performance (results not shown).

**Table 2:** Performance of different methods based on an independent dataset.

Methods	Sensitivity (%)	Specificity (%)	ACC (%)	MCC
All feature set	86.6	71.9	80.8	0.59
37-feature set	87.3	72.1	81.3	0.60
SIFT	79.5	71.3	76.3	0.51
PolyPhen-2	74.1	78.1	75.7	0.51
Bongo	21.6	84.7	46.6	0.09

*SIFT*, *PolyPhen-2*, *Bongo *and *SAPRED*, four well established methods, were used for benchmarking. With our independent dataset, *SIFT *[6] yielded a sensitivity of 79.5% and specificity of 71.3% while *PolyPhen-2 *yielded a sensitivity of 74.1% and specificity of 78.1% (Table 2). *Bongo *achieved a low sensitivity of 21.6% and specificity of 84.7%, which were similar with the results reported by Cheng *et al. *[39]. Using the dataset compiled by Ye *et al. *[21], our method achieved a sensitivity of 90.5% and specificity of 66.5% (*ntree *= 300 and *mtry *= 3). The corresponding ACC and MCC were 82.3% and 0.60, respectively. *SAPRED *achieved a higher sensitivity of 93.8% and a lower specificity of 61.3%. It yielded an ACC of 82.6% and MCC of 0.60. It should be noted that in this study, network features were introduced to depict an SAP instead of conventional structural features such as nearby functional sites, secondary structure, and hydrogen bonds. The satisfactory performance suggests that network features also include the information provided by structural features and this method can complement to the existing methods for predicting disease-associated SAPs.

## Discussion

From a biological viewpoint, mutual restraint of residues is crucial for the correct functioning of a proper structure[23]. Network topological features were adopted in the present study to describe both local and global residue interactions: *degree *and *clustering coefficient *were used for the former, and *closeness *and *betweenness *were used for the later. This can be understood from the fundamental aspects of protein structure. A special local structure is usually maintained by the cooperation of several residues. In this case, residues with more neighbors would naturally be more crucial in residue interactions [34], which would exert a greater influence on the local structure. In this sense, the frangibility upon residue substitution may be related to the density of the local structure. In biology, high *betweenness *is expected in the case of key residues that acting as a bridge in protein structure[41], such as those that bring together two different secondary structures. It was reported that *closeness *could indicate the functional role of a residue[40]. So, it is not surprising that high *closeness *values were observed for disease-associated SAPs. It is therefore reasonable to use these features to depict the structural/functional role of a residue.

Moreover, it was observed that the topologically important neighbors would likely be more conserved. It would be reasonable to expect that an SAP close to structural/functional key residues would more likely to be associated with diseases. This is why several studies have designed features to indicate the distance of an SAP to the function site [9,10,21]. In this sense, these network-based environmental features can depict the environment an SAP lies in.

We also compared network features with widely used conservation features. In contrast to conservation features, network features characterize the SAP in such a manner that its interactions with other residues in local/global protein structure are revealed. The performance of these features further proves their ability to distinguish disease-associated SAPs from polymorphic SAPs from the viewpoint of the roles of the focused residues in proteins. Moreover, the performance of the environmental feature set demonstrates that a dysfunctional mutation is closely correlated to the environment it lies in.

We compared our method with several well established approaches. The satisfactory performance of our method suggests that network features indicate the importance of a position in the context of the entire protein. It is therefore reasonable to believe that studying SAPs by analyzing residue interactions in a protein is both feasible and promising.

## Conclusions

Residues are in contact with each other, but their positions and conformations are restricted to ensure the maintenance of proper structure and function. Here, we represented a protein structure as a network, which allowed us to study the correlation between residues. Our results suggest that network topological features can appropriately reflect the role of a disease-associated SAP in both local and global structures by exploiting its correlation with other residues in a protein. The good performance obtained with the environmental feature set proves the feasibility of our method in detecting a disease-associated SAP by investigating the properties of its neighboring residues.

Several types of interactions are involved in a protein structure, including hydrophobic, hydrogen bond, van der Waal and electrostatic interactions. These may play specific roles in maintaining protein structure or function. It is still a challenge to feature such interactions in a protein structure network, although PSN has exhibited its advantage in revealing correlations between residues. It is anticipated that a PSN with more refined residue interactions should accurately reflect the structural/functional role of a residue in a protein. We will conduct further analysis in our future studies.

## Methods

### Data collection

We compiled an SAPs dataset from the Swiss-Prot variant page [3,4]. To construct protein structure networks, only variants that mapped to 3D structures were considered. Here, we extracted the protein structures of the wild-type from the ModSNP [3] on the EXPASY website. We then removed problematic structures with incorrect residue substitution or erroneous position record. The final dataset contained 6527 SAPs from 1094 proteins, including 3953 disease-associated and 2574 polymorphic SAPs, among which 127 proteins contained both disease-associated and polymorphic SAPs. An independent dataset was randomly selected, which consisted of 218 proteins with 696 disease associated and 456 polymorphic SAPs (see Additional file 1). It was used as a benchmark for evaluating our model as well as for comparing it with other published methods. The remaining 876 proteins with 3257 disease associated and 2118 polymorphic SAPs were used to perform 5-fold cross-validation (see Additional file 2).

### Random forest

The random forest package in R was employed for model training. The prediction models were provided in the additional files (see Additional file 3). The random forest is an ensemble classifier based on decision trees[42,43], which has been commonly used for classification and regression tasks. Two parameters, *ntree *and *mtry*, is crucial in this algorithm. *ntree *is the number of trees to grow and *mtry *is the number of variables selected to determine the decision at a node of the tree. In this study, they were optimized using a grid search approach. During the grid search, the optimal *ntree *and *mtry *were determined based on 5-fold cross-validation. The random forest package also offers a module for feature evaluation in which three measures are provided: selection frequency, Gini importance and permutation importance. In this study, we used the permutation importance to distinguish informative features from uninformative features. The estimation procedure was repeated 100 times, and the averaged values were used for this measurement. Here, sensitivity, specificity, total accuracy (ACC), and Matthew's correlation coefficient (MCC) were adopted for model evaluation. The Formula for each measure is listed as follow:

(1) $$Sensitivity=\frac{TP}{TP+FN}$$

(2) $$Specificity=\frac{TN}{TN+FP}$$

(3) $$Accuracy=\frac{TP+TN}{TP+FN+TN+FP}$$

(4) $$MCC=\frac{TP\times TN-FP\times FN}{\sqrt{\begin{array}{l} \left(TP+FP)(TP+FN\right)\\ \left(TN+FN)(TN+FP\right) \end{array}}}$$

where *TP *is the number of correctly predicted positive sample, *TN *is the number of correctly predicted negative sample, *FP *is the number of incorrectly predicted positive sample, and *FN *is the number of incorrectly predicted negative sample.

### Protein structure network

In a protein, residue interactions arise from covalent and/or non-covalent bonds between atoms. For convenience, the contacts are defined as follows: each residue is represented by the center of its side chain atom positions, but in the case of glycine, the C_α _is treated as the center. A contact is therefore identified on the condition that the distance between the centers of the two residues are within 6.5 Å [44]. Several topology features were derived from such networks.

### Feature extraction

Four network topological features were examined in this study.

*Degree *is the number of edges incident to a vertex. This is calculated as

(5) $$\delta (i)=\sum_{j\in N}a_{i,j}$$

where a_*i,j *_is the number of contacts between vertices *i *and *j*, and *N *is the set of total vertices. Within a protein structure, the δ(*i*) of a residue refers to its direct connectivity to other residues and is a non-negative integer value.

The *clustering coefficient *is a measure of the closeness the neighbors of a vertex. It can be defined as

(6) $$C(i)=\frac{2e_{i}}{\delta_{i}\left(\delta_{i}-1\right)}$$

where *e*_*i *_is the virtual and δ_*i*_(δ_*i*_-1)/2 is the maximum possible number of edges between the neighbors of vertex *i*.

*Closeness *is a centrality measure of a vertex and is defined as the average geodesic distance to all other vertices. It can be calculated as-

(7) $$CC(i)=\frac{N-1}{\sum_{i\neq j}d_{i,j}}$$

where N is the total number of vertices and d_*i,j *_is the shortest path between vertices *i *and *j*. The closeness score indicates the status of a residue in the entire protein structure.

*Betweenness *refers to how often a vertex occurs on the shortest paths between other vertices. It can be calculated as

(8) $$B_{i}=\sum_{j,k\in N,j\neq k}\frac{n_{j,k}\left(i\right)}{n_{j,k}}$$

where *n*_*j,k *_is the number of all geodesics linking vertices *j *and *k*. The term *n*_*j,k*_(*i*) indicates the number of shortest paths connecting *j *and *k *passing through vertex *i*. *Betweenness *is sensitive to the protein length. To avoid the bias, the feature was scaled by the protein length. This parameter was reported to performed well in identifying the hot spots in protein interactions[41]. For more detailed descriptions of these parameters, please refer to Newman and Watts[45,46].

Other sequence and structural features employed in this study include sequence conservation, point accepted mutation (PAM) 250, solvent accessible area, aggregation propensities and HLA family. Solvent accessibility was reported to be an important feature in SAP prediction. We derived the solvent accessible area of each residue by using DSSP[47].

Position-specific iterated BLAST (PSI-BLAST) [48] has been generally used in studies on proteomics. In this study, it was implemented against the Swiss-Prot database with an E-value cutoff of 1E-3 and 3 iterations. The output position-specific scoring matrix (PSSM) and weighted observed percentage for both wild-type and variant as well as their differences were taken to characterize a mutation. Furthermore, the conservation score of an SAP site can be defined as

(9) $$Score_{i}=-\sum_{j=1}^{20}p_{i,j}\log_{2}p_{i,j}$$

where *p*_*i,j *_is the frequency of amino acid *j *at position *i*. A lower value suggests lower entropy (more conserved) at a position and vice versa.

In our method, probability of the substitution according to PAM250 was taken to score a mutation. For a more detailed description please refer to Dayhoff *et al. *[49]. In previous studies, aggregation propensity was thought to be a significant factor in disease susceptibility[30]. Therefore, this feature was adopted here. Aggregation propensities for wild-type and variant amino acids were taken from TANGO[50]. The aggregation propensity change for a fragment upon a single variant was also taken into account. Moreover, by following the approach described by Ye *et al. *[21], a feature was employed to determine whether a protein in which an SAP is located belongs to the HLA family.

Based on the structure network, neighboring residues were extracted as those with direct contacts with an SAP, i.e., those with a distance to the focused residues that was no more than 6.5 Å. We investigated the properties of neighboring residues in terms of network topological features and conservation scores. Our analysis indicated that, the five most conserved neighboring residues can appropriately reflect the environment around a mutation. For SAP sites with less than five neighbors, zeros were added. Thus, the environmental feature of a SAP site could be encoded by a 25-dimensioned vector.

## Availability and requirements

Project name: NetSAP;

Project home page: http://cic.scu.edu.cn/bioinformatics/NetSAP.zip;

Operating system(s): Linux and Microsoft Windows;

Programming language: R language (Version 2.7.2);

License: None

## Abbreviations

SNP: single nucleotide polymorphisms; SAP: non-synonymous single nucleotide polymorphisms; PSN: protein structure network.

## Authors' contributions

YL wrote the program, designed the experiments, and drafted the manuscript. YL, ZW, and JX helped in the analysis and discussion. ZW and HY refined the manuscript and provided useful comments. ML initiated and supervised the entire project. All authors read and approved the final manuscript.

## Supplementary Material

Additional file 1 **The independent dataset used in this study**. Click here for file

Additional file 2 **The dataset used for training the prediction model**. Click here for file

Additional file 3 **The models for predicting disease-associated SAPs**. Click here for file
